# Abnormal characteristics of intestinal microenvironment in HIV immunological non-responders

**DOI:** 10.3389/fimmu.2026.1796163

**Published:** 2026-04-14

**Authors:** Huanxia Liu, Yanguang Yang

**Affiliations:** 1 Department of Infectious Disease, Public Health Clinical Center of Chengdu, Chengdu, China; 2 School of Basic Medical Sciences, Chengdu University of Traditional Chinese Medicine, Chengdu, China

**Keywords:** CD4^+^ T cells, HIV, HIV-infected immunological non-responders, intestinal immune, intestinal microenvironment

## Abstract

Acquired immunodeficiency syndrome (AIDS) is one of the most dangerous diseases threatening global public health. Antiretroviral therapy (ART) is currently the primary treatment for people living with HIV (PLWH). However, some patients are classified as immunological non-responders (INRs), defined by the failure to achieve adequate CD4^+^ T cells reconstitution despite continuous viral suppression, and are associated with inferior clinical outcomes. This behavior may be linked to the ongoing dysfunction of the intestinal microenvironment. Although PLWH exhibit similar clinical changes such as intestinal mucosal injury, barrier failure, and microbial community problems, intestinal microenvironment abnormalities in INRs are more severe. The specific manifestations include persistently low levels of intestinal CD4^+^ T cells with limited reconstitution, along with a significant reduction in the proportion of Th17 cells, leading to severe impairment of mucosal anti-infective capacity and immune regulatory function. Additionally, elevated levels of pro-inflammatory mediators drive chronic inflammation, thereby exacerbating tissue damage. Furthermore, microbial dysbiosis is more pronounced, characterized by a marked decrease in beneficial symbiotic bacteria and an expansion of opportunistic pathogens. In contrast, immunological responders showed some degree of recovery in these indicators. These pathological features are not only associated with a higher risk of disease progression and complications in INRs but also provide a theoretical basis for developing adjuvant treatment strategies targeting intestinal immune reconstitution. In addition, we summarize the current mainstream definitions of INRs and propose a more robust definition. This review systematically elaborates the pathogenic mechanisms and potential intervention strategies underlying intestinal microenvironment abnormalities in INRs and holds important clinical value for improving the long-term prognosis of patients and advancing individualized treatment.

## 1 Introduction

Acquired Immunodeficiency Syndrome (AIDS) is caused by the human immunodeficiency virus (HIV). According to recent reports, there were approximately 1.3 million new HIV infections worldwide in 2024, highlighting that HIV/AIDS remains a major global public health concern (1). Since 2010, the proportion of people living with HIV (PLWH) receiving antiretroviral therapy (ART) has increased to 77% globally (1). The broad deployment of ART has resulted to a 54% drop in annual new HIV infections and a 40% reduction in AIDS-related fatalities compared to previous levels (1). Despite these gains, a significant proportion of patients experience inadequate CD4^+^ T cell recovery after starting ART, a disease known as immunological non-responder (INRs), with a frequency ranging from 10% to 40% (2). Individuals with INRs face a significantly elevated risk of opportunistic infections, cancer, and death (2, 3). This impaired immune reconstitution may be attributable to profound dysfunction of the intestinal immune system. Damage to gut-associated lymphoid tissue (GALT), a crucial regulator of systemic immune homeostasis, may be specifically involved (4, 5).

Under normal conditions, the gut microbiota maintains a relatively stable equilibrium. However, HIV infection damages intestinal mucosal immunity, alters gut microbial diversity and composition, thereby driving systemic inflammation and impairing CD4^+^ T cells recovery (6–8). Additionally, PLWH experience significant alterations in the gastrointestinal microenvironment and immune system, which result in increased intestinal mucosal permeability, particularly elevated levels of intestinal fatty acid-binding protein (I-FABP) and lipopolysaccharide (LPS), and encourage the release of pro-inflammatory cytokines (9, 10). More critically, massive depletion of CD4^+^ T cells occurs in the intestinal mucosa, with T helper (Th) 17 cells bearing a memory phenotype exhibiting the most pronounced loss in INRs (11, 12). This facilitates microbial translocation and persistent immune activation, thereby accelerating disease progression (13, 14).

HIV infection leads to a reduction in the diversity and abundance of the gut microbiota, thereby impairing the recovery of host immune function (15, 16). Furthermore, the fecal microbial α-diversity in PLWH was significantly decreased, and the proportion of potential pathogenic bacteria in the intestinal flora was markedly increased, including *Proteobacteria*, *Enterococcus*, and *Streptococcus* (17). Despite ART, intestinal microbial diversity remains incompletely restored. Moreover, the metabolic processes of ART drugs may also exert adverse effects on the composition of the gut microbiota (18). Notably, INRs exhibit more severe intestinal dysbiosis compared to immunological responders (IRs), characterized by an increase in pathogenic bacteria and a decrease in beneficial taxa (5). This disruption in intestinal microecology may represent a key mechanism contributing to suboptimal ART efficacy in INRs. Therefore, targeted regulation of intestinal immunity holds promise as a strategy to enhance ART outcomes in this population. This review summarizes current definitions of INRs, characterizes the intestinal mucosal immune abnormalities that underpin their impaired CD4^+^ T cells recovery, and outlines mechanistically grounded intervention strategies, thereby strengthening the rationale for improving long-term prognosis and advancing individualized management.

## 2 Definition of INRs

INRs refer to a clinical state in which CD4^+^ T cells recover incompletely after PLWH receive ART, despite continued suppression of viral load. However, their definition and diagnostic criteria have not been unified across different studies (**Table 1**). It is generally believed that after 2 to 7 years of ART treatment, if the plasma viral load is below the lower limit of detection while the CD4^+^ T cell counts remain below 350/μL, patients may be classified as INRs. Such patients face a higher risk of disease progression and are prone to opportunistic infections, major non-AIDS-defining events, and even death.

**Table 1:** Definitions of INRs in different studies.

Definitions of INRs	Number of samples	Countries/ areas	Year	Ref.
ART>1 years, CD4^+^ T cell<200/μL	44	Italy	2011	(19)
During ART, CD4^+^ T cell percentages <30%	146	Brazil	2018	(20)
ART>1 years, CD4^+^ T cell<250/μL	30	India	2020	(21)
ART>4 years, CD4^+^ T cell<400/μL	39	Norway	2021	(22)
ART≥2 years, CD4^+^ T cell<350/μL	28	Canada	2021	(129)
ART>4 years, CD4^+^ T cell<350/μL	–	China	2024	(2)
ART≥2 years, CD4^+^ T cell<350/μL	1895	Netherlands	2024	(23)
ART>3 years, CD4^+^ T cells increased by at least 200/μL.	100	Spain	2024	(24)
ART for 2–14 years, CD4^+^ T cell<350/μL	131	India	2025	(25)
ART>1 years, CD4^+^ T cell<350/μL	40	Indonesia	2025	(130)

ART, antiretroviral therapy; INRs, immunological non-responders.

**Figure 1:**
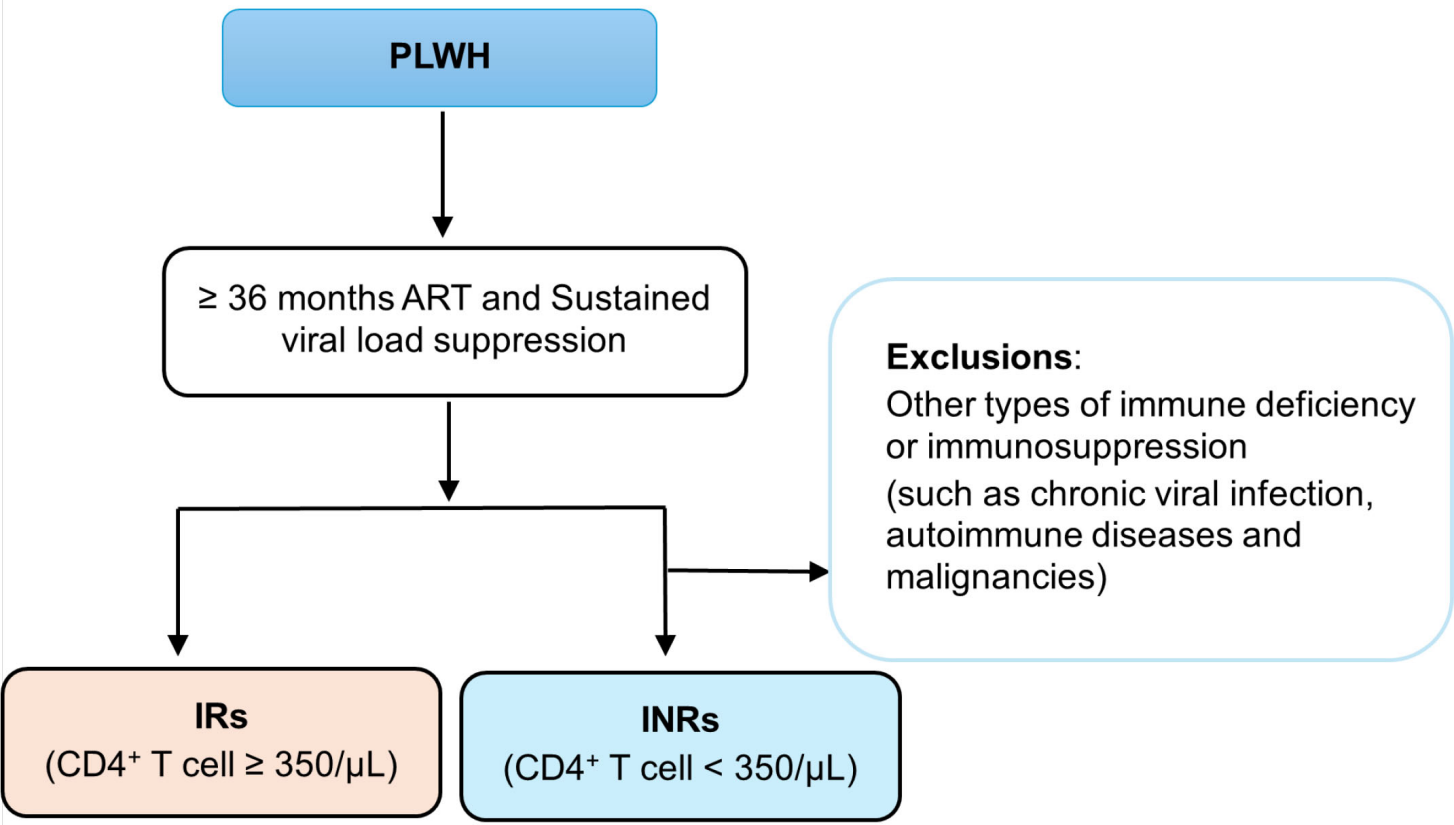
Proposed operational definition criteria for immunological non-responders (INRs) in people living with HIV (PLWH).

In 2011, Italian research institutions defined INRs as individuals who had received ART for more than one year and whose CD4^+^ T cell counts remained below 200/μL (19). In 2018, a Brazilian study proposed that the criterion for INRs be defined as a CD4^+^ T cells percentages below 30%, measured on two consecutive occasions during ART treatment (20). However, the criteria for identifying INRs were receipt of ART for at least one year and a CD4^+^ T cells counts below 250/μL, as proposed in 2020 (21). In 2021, researchers in Norway proposed that INRs be defined by more than four years of ART, a CD4^+^ T cell counts below 400/μL, and sustained suppression of HIV RNA in blood for over 3.5 years (12, 22). According to the China HIV/AIDS Diagnosis and Treatment Guidelines, the diagnostic criteria for INRs were: receipt of ART for more than 4 years, sustained viral load suppression for more than 3 years, and a persistently low CD4^+^ T cells count below 350/μL. At the same time, other factors that may lead to long-term CD4^+^ T cells decline must be excluded (2). Such conditions include known immunodeficiency disorders, immunosuppressive conditions, chronic viral infections, hematologic neoplasms, and long-term use of immunosuppressive drugs. The criteria for defining INRs in a clinical trial conducted in the Netherlands in 2024 include receipt of ART for at least two years and a CD4^+^ T cell counts below 350/μL (23). Simultaneously, Spanish studies defined INRs as individuals receiving at least three years of ART and exhibiting a CD4^+^ T cell counts increase of less than 200/μL (24). Interestingly, a recent article showed that the criteria for identifying INRs in India were long-term ART lasting 2–14 years and a CD4^+^ T cells count below 350/μL (25). Finally, some researchers defined NRs as individuals whose CD4^+^ T-cell count was below 500/μL prior to ART initiation, remained below 500/μL after two years of ART, and exhibited an absolute CD4^+^ T cells increase of less than 200/μL over this period (26). Interestingly, the CD4/CD8 ratio was also used to define INRs. Specifically, after two years of ART, a CD4/CD8 ratio greater than 1 indicated IRs, whereas a ratio less than 1 indicated INRs (27). However, there remains a lack of standardized criteria from international authoritative organizations. In summary, based on current research findings, assessment of INRs after ART should primarily rely on the current CD4^+^ T cells counts. Based on the above studies, this review recommends that the definition of INRs be clearly specified as individuals who have received standardized ART for at least 36 months, maintain sustained virological suppression, and have a CD4^+^ T cells count below 350/μL. At the same time, a systematic evaluation must be conducted to rule out other confounding factors that may contribute to persistent CD4^+^ T cells decline, such as chronic viral infections, autoimmune diseases, and malignancies (**Figure 1**).

## 3 Overview of the intestinal microbiota

A large number of microorganisms inhabit the human intestine, forming a complex microbial ecosystem (28). As a potential hidden organ, the gut microbiota maintains long-term symbiosis with its host and primarily consists of diverse microbial groups such as bacteria, fungi, and viruses, as well as dominant bacterial phyla including *Firmicute*s, *Bacteroidetes*, and *Actinobacteria* (29). These microorganisms colonize the intestinal lumen and maintain a delicate dynamic balance with intestinal epithelial cells. This equilibrium not only effectively prevents pathogen invasion but also actively participates in regulating local and systemic immune responses (30). As the largest microecosystem in the human body, the intestinal microbiota sustains a symbiotic relationship with the intestinal mucosa, providing the host with essential metabolic, immune, and intestinal protective functions (31–33). It also maintains the integrity of the intestinal mucosal barrier and inhibits the colonization of pathogenic microorganisms, thereby playing a crucial role in maintaining host health and homeostasis. Normal intestinal microbiota produces short-chain fatty acids (SCFAs), such as acetic acid, propionic acid, and butyric acid, which serve as energy sources for intestinal epithelial cells and exert anti-inflammatory and immunoregulatory effects (34–36). Furthermore, the normal microbiota can inhibit the overgrowth of potential pathogens by occupying ecological niches and secreting antimicrobial substances, thus preserving intestinal microbial stability (37). In recent years, with the continuous advancement of high-throughput sequencing technologies and growing research depth, the mechanistic roles of intestinal microorganisms in PLWH and its related complications have become a major research focus.

## 4 Abnormal characteristics of the gut microbiota in INRs

**Figure 2:**
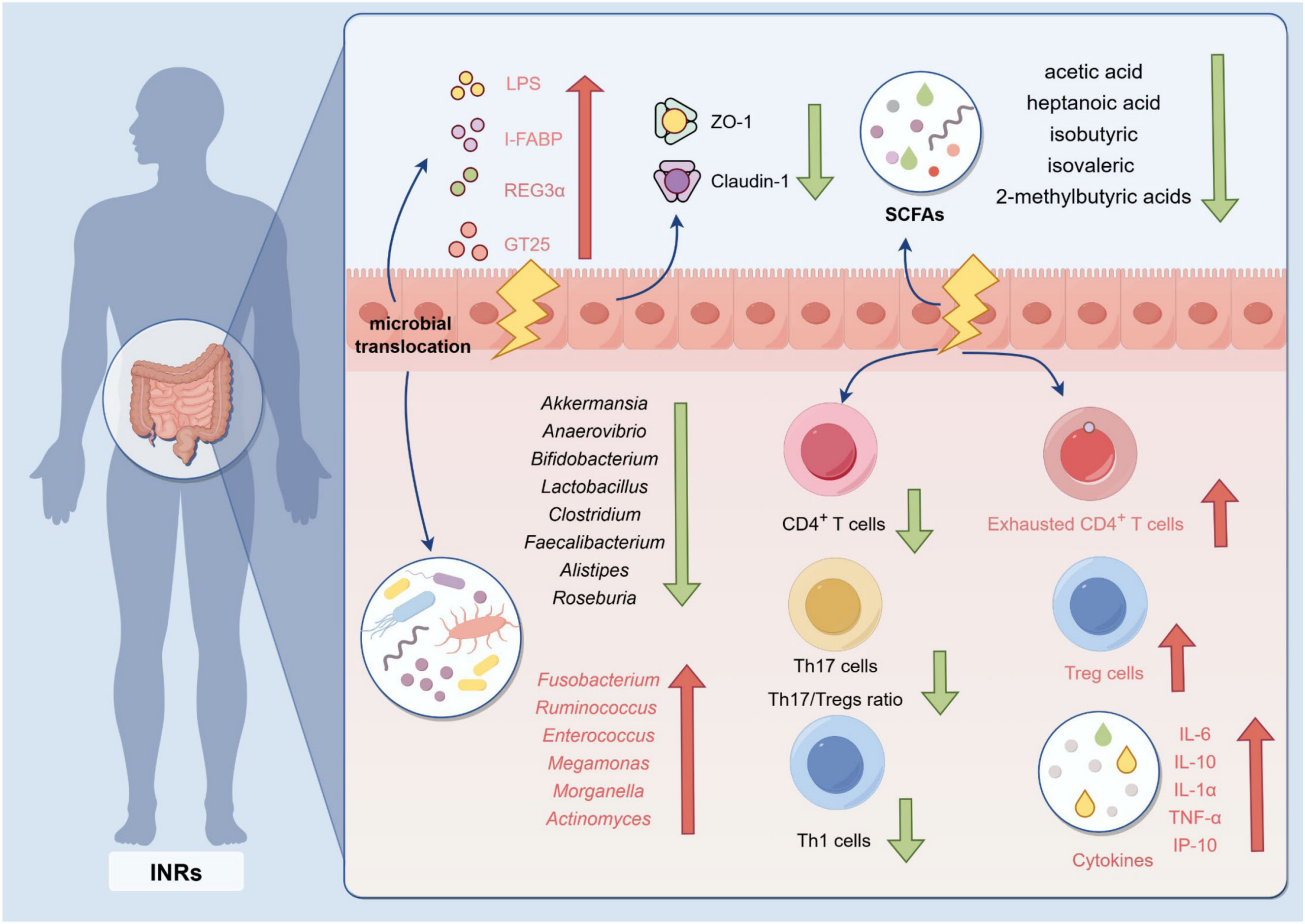
Abnormal characteristics of intestinal microenvironment in INRs, immunological non-responder Increased microbial translocation leads to increased intestinal permeability in individuals with INRs, which in turn causes intestinal mucosal epithelial damage; LPS, levels of its markers—lipopolysaccharide, I-FABP, intestinal fatty acid–binding protein , and REG3α, regenerating islet-derived protein 3α—increase significantly and trigger the release of pro-inflammatory cytokines (IL-6, IL-10, IL-1α, TNF-α, and CXCL10), driving persistent chronic inflammation. At the same time, the expression levels of key intestinal tight junction proteins ZO-1 and claudin-1 are significantly downregulated. More importantly, the structure of the intestinal microbiota is disrupted: the abundance of beneficial bacteria (such as *Akkermansia*, *Anaerovibrio*, *Bacteroides*, *Lactobacillus*, *Clostridium*, *Faecalibacterium*, *Alistipes*, and *Roseburia*) is reduced, while the enrichment of conditionally pathogenic bacteria (such as *Fusobacterium*, *Ruminococcus*, *Enterococcus*, *Megamonas*, *Morganella*, and *Actinomyces*) leads to reduced synthesis of SCFAs, short-chain fatty acids, including acetic acid, heptanoic acid, isobutyric acid, isovaleric acid, and 2-methylbutyric acid. Moreover, the absolute counts of CD4^+^ T cells, Th1 cells, and Th17 cells are significantly decreased, whereas the frequencies of exhausted CD4^+^ T cells and regulatory T cells (Tregs) are markedly elevated, leading to a pronounced Th17/Treg ratio imbalance that further disrupts intestinal microbial homeostasis.

The human intestine is the site with the highest density of lymphocytes, lymphoid tissue, and lymph nodes. Studies have demonstrated that the gut is not only one of the earliest and most severely affected target organs in HIV infection but also a critical site influencing the success of immune reconstitution. Since the main target cells of HIV infection are lymphocytes, the intestine has become a highly virus-rich area and is considered a major viral reservoir for HIV (38, 39). Studies have demonstrated that the levels of HIV viral transcripts are elevated in INRs (24, 40, 41). Quantitative PCR analyses further confirmed that the size of the HIV reservoir in peripheral blood is significantly greater in INRs than in IRs, indicating a larger latent HIV reservoir in the former (40). Consistent with this finding, INRs exhibit heightened activation of the type I interferon signaling pathway and more pronounced CD4^+^ T cells depletion. Collectively, these pathological features suggest an increased risk of disease progression and a poorer clinical prognosis in INRs. The long-term persistence of viral reservoirs is one of the major obstacles to achieving a functional cure for HIV/AIDS. After HIV infection, the composition, structure and diversity of the intestinal microbiota have changed significantly, especially a decrease in the abundance of SAFAs-producing bacteria (such as *Roseburia* and *Clostridium*), suggesting that infected individuals already exhibit intestinal dysbiosis and an increased risk of impaired mucosal barrier function (42). More critically, in the early stages of HIV infection, a large number of CD4^+^ T cells are destroyed in the intestinal mucosa, with the most significant decrease observed in Th17 cells exhibiting a memory phenotype (43, 44). Th17 cells play a central role in maintaining the integrity of the intestinal epithelial barrier, resisting colonization by pathogenic microorganisms, and regulating local immune responses. A large loss of Th17 cells can increase intestinal epithelial permeability, promote the translocation of microorganisms and their metabolites (such as LPS) from the intestinal lumen into the bloodstream, and ultimately trigger sustained systemic immune activation and chronic inflammation (45, 46). Importantly, intestinal immune dysfunction in INRs was more pronounced (**Figure 2**). Specifically, the number of CD4^+^ T cells in the gut remained persistently low with limited recovery, leading to severe impairment of mucosal anti-infective capacity and immune regulatory function (4, 47). Furthermore, INRs were associated with elevated levels of pro-inflammatory cytokines, which drove a chronic inflammatory state and exacerbated systemic immune activation (47). Concurrently, microbial imbalance became increasingly evident, characterized by a reduction in beneficial bacteria and an expansion of opportunistic pathogens. This process plays a critical role in the progression of INRs.

### 4.1 Intestinal bacteria in INRs

In the early stages of HIV infection, the virus rapidly targets and destroys GALT, particularly the intestinal mucosal immune microenvironment enriched with CD4^+^ T cells, leading to widespread immune cell apoptosis and disruption of intestinal barrier integrity (48, 49). This pathological process not only severely compromises the host’s defense against pathogens but also disrupts the dynamic equilibrium between the gut microbiota and the host, resulting in marked intestinal dysbiosis and thereby establishing a critical pathological foundation for subsequent chronic immune activation and microbial translocation (50, 51). In contrast to the diminished abundance of beneficial dominant phyla such as Bacteroidetes and Firmicutes, a relative expansion was observed in pro-inflammatory or opportunistic taxa, including *Prevotellaceae*, *Proteobacteria*, and *Fusobacteria* (52, 53). Another study found that PLWH exhibited a higher relative abundance of *Prevotella* and a lower relative abundance of *Bacteroides* in the gut microbiota compared to healthy controls (54). Compared with healthy controls, the relative abundances of *Fusobacterium*, *Ruminococcus gnavus*, and *Megamonas* were significantly increased in the IRs and INRs groups, whereas those of *Faecalibacterium*, *Alistipes*, *Bifidobacterium*, *Eubacterium rectale*, and *Roseburia* were significantly reduced (55). Furthermore, beneficial commensal genera such as *Akkermansia*, *Anaerovibrio*, *Bifidobacterium*, and *Clostridium* were also reduced to varying degrees (56). Overall, the gut microbiota in PLWH showed a decrease in beneficial bacteria and an increase in pathogenic bacteria. Among these, *Akkermansia muciniphila* (*A. muciniphila*), a key probiotic species, can specifically colonize the colonic mucus layer, promote mucin production, enhance epithelial integrity, maintain barrier function, and exert immunomodulatory and anti-inflammatory effects through mechanisms including regulation of the Th17/regulatory T (Treg) cells balance (57, 58). Multiple studies have consistently reported that the abundance of *A. muciniphila* is significantly lower in PLWH compared to healthy controls (56, 59, 60). Therefore, supplementation with beneficial bacterial strains such as *A. muciniphila* to restore their intestinal colonization represents a promising adjuvant strategy for improving gut microbial homeostasis and immune function in PLWH.

As a key regulator of host immune homeostasis, the gut microbiota plays a significant role in the pathogenesis and progression of INRs. When the CD4^+^ T cell counts in peripheral blood fell below 350/μL in PLWH, intestinal microbiota dysbiosis was further exacerbated, characterized by reduced microbial diversity, decreased abundance, altered distribution, and significant changes in metabolite levels. It has been reported that there is no significant difference at the phylum level between individuals with IRs and those with INRs (55). The main manifestation was that the relative abundance of *Escherichia-Shigella* and *Blautia* in IRs was higher than that of INRs. At the genus level, the abundance of *Faecalibacterium* was reduced in the intestines of INRs, whereas that of *Alistipes* was increased (61). Interestingly, *Ruminococcaceae* and *Alistipes* were positively correlated with current CD4^+^ T cell counts (55). The observed discrepancies in the results may stem from variations in the definition criteria for INRs across studies. However, patients in the INRs group exhibited higher abundances of *Faecalibacterium prausnitzii*, unclassified *Subdoligranulum* sp., and *Coprococcus comes* compared to those in the IRs group at the species level (54). Importantly, the relative abundances of *Subdoligranulum* sp. and *Coprococcus comes* showed significant positive correlations with peripheral CD8^+^HLA-DR^+^ T cell counts. The expansion in both the absolute number and frequency of CD8^+^ T cells represents a hallmark immunological feature of HIV infection, particularly within the activated subset co-expressing HLA-DR and CD38 (62). Interestingly, INRs consistently lacked the probiotic *Lactobacillus* both prior to and following ART (19). A recent study reported that in healthy controls, the relative abundances of gut microbiota at the family level, including *Lachnospiraceae* and *Ruminococcaceae*, and at the genus level, including *Coprococcus*, *Lachnoclostridium*, *Ruminococcus torques*, *Ruminococcus*, *Faecalibacterium*, and *UBA1819*, were significantly higher than those observed in IRs and INRs (4). Similarly, in another study, *Enterococcus* increased in the gut of INRs, while *Ruminococcus_gnavus_group* decreased (63). Notably, the relative abundance of these microbial taxa was significantly lower in INRs than in IRs (4). In another study, the relative abundances of *Actinomycetales*, *Micrococcaceae*, *Actinomyces*, *Intestinibacter*, and *Rothia* were elevated in INRs than in IRs, whereas *Sutterellaceae*, *Parabacteroides*, *Veillonella*, and *Butyricimonas* exhibited lower relative abundances in INRs relative to IRs (5). Additionally, *Morganella*, a conditional pathogen, was present exclusively in the INRs group with higher relative abundance and was absent in the IRs group (42, 64). Glycosyltransferase 25 (GT25) was significantly expressed in the INRs group but was undetectable or expressed at extremely low levels in the IRs group (42). It is worth noting that correlation analysis showed a positive correlation between the relative abundance of *Morganella* and the GT25 secretion level. Therefore, the abnormal increase in *Morganella* may be associated with incomplete immune reconstitution after ART and may mediate this pathological process through GT25 secretion. Taken together, *Morganella* is expected to serve as a potential biomarker for predicting persistent inadequate immune reconstitution in patients with INRs receiving ART. Compared with research on the intestinal microbiota, there are few studies on the oral microbiota of INRs. One study showed that *Proteobacteria* in the mouths of INRs were more abundant than those in IRs (65). To sum up, the composition and relative abundance of intestinal microbiota are closely associated with the occurrence of INRs.

### 4.2 Intestinal barrier in INRs

Chronic HIV infection can cause significant structural damage to the intestinal tissues of patients, characterized by atrophy of the intestinal epithelial villi, impaired absorptive function, focal epithelial degeneration, and extensive apoptosis of the intestinal mucosa and associated immune cells (9, 66, 67). Concurrently, the expression of tight junction proteins (such as ZO-1, occludin, and claudin) is downregulated, resulting in significant impairment of intestinal barrier function and increased gut permeability (68). This barrier disruption not only exacerbates local intestinal inflammation but also increases intestinal permeability, thereby triggering gut microbiota dysbiosis, commonly observed in PLWH as reduced microbial load and decreased alpha diversity (43, 53, 69). Additionally, there are varying degrees of intestinal tissue damage in PLWH, and the extent of intestinal damage is positively correlated with HIV DNA levels and negatively correlated with peripheral blood CD4^+^ T cell counts. After ART, intestinal injury in both INRs and IRs did not fully resolve. However, mucosal repair was more pronounced in IRs, whereas INRs exhibited persistent and severe intestinal damage, with evidence of mucosal tissue shedding. This indicates that intestinal injury is more severe in INRs than in IRs. It was further found that the expression level of Claudin-1 and ZO-1 in the intestinal epithelial tissues of INRs was significantly decreased, whereas the expression levels of Caspase-3 and tumor necrosis factor-α (TNF-α) were elevated (70, 71). These results indicate increased intestinal epithelial permeability, aggravated tissue damage, concurrent glandular cell apoptosis, and a systemic inflammatory response. An imbalance in the intestinal microbiota can further compromise intestinal barrier function and alter the production of microbial metabolites (such as SCFAs). SCFAs serve as critical signaling molecules that regulate intestinal immune homeostasis and maintain epithelial barrier integrity (72). A study has shown that the relative abundance of *Bacteroidetes* is significantly enriched in the intestines of simian immunodeficiency virus–infected nonhuman primates, an increase that may compromise SCFA production and thereby disrupt intestinal barrier integrity (73). Therefore, a strong correlation exists between INRs and intestinal barrier function, with the intestinal microbiota potentially serving as a key mediator in this relationship.

### 4.3 Intestinal metabolites in INRs

LPS are a major component of the cell walls of Gram-negative intestinal symbionts and opportunistic pathogens. When intestinal mucosal barrier function is compromised, LPS can translocate from the intestinal lumen to the portal vein and systemic circulation (a process termed microbial translocation) (74). In PLWH, early depletion of gut-associated lymphoid tissue increases intestinal epithelial permeability, enabling substantial LPS leakage across the physical barrier into peripheral blood, leading to persistent activation of the innate immune system (75, 76). LPS binds to Toll-like receptor 4 (TLR-4) on monocytes and macrophages, activating the NF-κB signaling pathway and inducing the release of pro-inflammatory cytokines such as interleukin-6 (IL-6), TNF-α, and interleukin-1β (IL-1β), thereby initiating and sustaining chronic immune activation. This state is recognized as one of the key drivers of HIV disease progression and impaired immune reconstitution. In a prior study, plasma concentrations of LPS and soluble CD14 (sCD14), a key co-receptor involved in LPS-mediated immune activation, were significantly elevated in PLWH compared to healthy controls, indicating persistent microbial translocation (19). However, no significant differences were observed between IRs and INRs, either before the initiation of ART or one year after treatment, suggesting that these markers alone may not reliably distinguish immunological response trajectories despite ongoing systemic inflammation (19). However, overall levels of microbial translocation were markedly lower in IRs and healthy controls compared to INRs, a difference primarily attributable to elevated concentrations of LPS and total bacterial 16S rDNA (77). This also suggests that INRs may suffer more severe intestinal barrier damage and persistent translocation of microbial products into the bloodstream, thereby triggering a heightened systemic inflammatory response, which contributes to reduced ART efficacy and poorer clinical outcomes. The inconsistency in LPS testing results between the two studies may be due to differences in diagnostic criteria for INRs: the former is defined as CD4^+^ T cell counts still below 200/μL one year after receiving ART, while the latter is defined as CD4^+^ T cell counts below 350/μL eight years after treatment. The heterogeneity of diagnostic criteria significantly affects the comparability of study results, so there is an urgent need to establish globally recognized diagnostic criteria for INRs to promote standardized integration of clinical data and cross-study comparisons. Notably, sCD14 levels did not differ significantly between IRs and INRs, a finding consistent with previously reported studies (19, 77). Interestingly, LPS levels were positively correlated with the relative abundance of *Ruminococcus* and *Fusobacterium*, but negatively correlated with the relative abundance of *Faecalibacterium* (55). *Faecalibacterium* is regarded as a next-generation probiotic bacterium, whose relative abundance is reduced in various diseases, including inflammatory bowel disease, dermatitis, depression, and other gastrointestinal disorders (61, 78). Therefore, LPS translocates into the bloodstream in INRs, contributing to chronic inflammation, which may be associated with a reduced abundance of *Faecalibacterium* in the gut. Measuring plasma LPS levels may thus serve as a valuable tool for evaluating the extent of intestinal barrier dysfunction and the degree of chronic immune activation in INRs, thereby providing a potential biomarker foundation for risk stratification and the development of targeted therapeutic interventions.

The intestinal microbiota produces key metabolites known as SCFAs, which are primarily composed of acetic, propionic, and butyric acids (79). Their concentrations are influenced not only by dietary patterns and the composition of the gut microbial community, but also by host immune function and the maintenance of intestinal homeostasis (35, 36). These metabolic intermediates serve as a major energy source for colonic epithelial cells and play critical roles in modulating host immune responses, strengthening intestinal barrier integrity, and suppressing pathogen colonization (80, 81). HIV depletes GALT early in infection, resulting in impairment of the intestinal mucosal barrier, increased microbial translocation, and chronic immune activation. These pathological changes subsequently lead to a marked reduction in the abundance of SCFA-producing beneficial bacteria, ultimately compromising intestinal integrity and function (82, 83). Similarly, the relative abundance of key functional genes involved in SCFAs production has significantly decreased (56, 84). Similarly, due to the reduced abundance of SCFA-producing bacteria in INRs, such as *Lachnospiraceae* and *Ruminococcaceae*, intestinal SCFAs concentrations are significantly decreased, particularly acetic acid and heptanoic acid (4). Besides, the fecal concentrations of isobutyric, isovaleric, and 2-methylbutyric acids in IRs were higher than those in INRs, but no differences were detected in serum samples (27). However, differences in SCFA profiles between IRs and INRs have also been reported. The primary reason for these contradictory results is the lack of standardized diagnostic criteria for INRs. Therefore, restoring SCFAs levels in INRs may be an effective strategy to shift immune non-response to immune response, thereby improving adverse outcomes in INRs. To summarize, the reduced capacity for intestinal SCFA production in PLWH is not only a biomarker of disease progression, but it may also be a key factor influencing the efficacy of ART, necessitating special attention in clinical care.

### 4.4 Intestinal immune cell in INRs

In the peripheral blood of healthy individuals, T lymphocytes account for the majority of all lymphocytes and constitute the main lymphocyte population; however, most lymphocytes in the body are distributed primarily in the GALT (85). Among the lymphocytes in the GALT, CD4+ T cells constitute the dominant subset within the T cell population. Acute HIV-1 infection targets intestinal lymphoid tissue early in the disease course, and robust viral replication leads to marked depletion of CD4+ T cells in the GALT (13, 86, 87). Compared with the decline in peripheral blood CD4^+^ T cells, the loss in intestinal lymphoid tissue occurs earlier and is more severe. Although effective ART can potently suppress viral replication, thereby reducing or preventing CD4^+^ T cell destruction and leading to a significant rebound in peripheral CD4^+^ T cell counts, recovery of intestinal CD4^+^ T cells remains limited (88). Furthermore, most gut-resident CD4^+^ T cells exhibit a memory phenotype and are enriched in effector T cells expressing high levels of C-C motif chemokine receptor 5 (CCR5), features that distinguish them from their peripheral blood counterparts (89, 90). Despite the profound depletion of gut-resident CD4^+^ T cells during chronic HIV infection and their incomplete restoration following ART, the dynamic changes in immune cells within intestinal tissues—particularly the mucosa—and their clinical implications remain poorly understood due to a lack of systematic and mechanistic investigation (91).

#### 4.4.1 CD4^+^T cells in INRs

According to their functional properties, CD4^+^ T cells can be classified into distinct subsets, including Th1, Th2, Th17, and Treg cells, each defined by unique cytokine profiles and immunological functions (92). Among these subsets, Th17 cells are a critical component of the intestinal mucosal immune system, constituting approximately 80%–90% of CD4^+^ T cells in the GALT (92). Th17 cells highly express CCR5 and CXCR4, the coreceptors essential for HIV entry, making them preferential targets for viral infection (44, 93). Furthermore, they harbor high levels of HIV dependency factors that support viral replication and express surface molecules such as CCR6 and integrin α4β7, which facilitate cell-to-cell transmission of HIV (13, 44). Consequently, Th17 cells exhibit heightened susceptibility to HIV infection; indeed, both R5-tropic and X4-tropic HIV strains have been shown to efficiently infect Th17 cells under *in vitro* culture conditions (94). The balance between Th17 and Treg cells is crucial for maintaining intestinal immune homeostasis (95). Chronic HIV infection leads to selective and extensive depletion of Th17 cells, thereby disrupting this equilibrium. In healthy individuals, the Th17/Treg ratio typically ranges from 1.0 to 1.2, but it declines markedly to between 0.75 and 0.2 in the advanced stages of chronic HIV infection (96). This diminished ratio is strongly associated with increased microbial translocation due to compromised mucosal integrity and impaired antiviral immune responses, ultimately undermining the host’s capacity to control pathogen dissemination.

INRs, a severe clinical phenotype observed during treatment of PLWH, are characterized by profound immune dysfunction. One study revealed that, in colonic mucosal biopsy samples, INRs exhibited a lower proportion of CD4+ T cells and elevated levels of interleukin-22 (IL-22) compared to IRs, along with a significantly higher proportion of IL-17-producing CD4+ T cells (Th17 cells). Notably, the increased number of Th17 cells was positively correlated with circulating levels of regenerating islet-derived protein 3 alpha (REG3α) and I-FABP, biomarkers of intestinal epithelial damage, suggesting a link between Th17 cell expansion and impaired mucosal integrity in INRs (12). Moreover, a study reported that INRs exhibit significantly lower counts of Th17 cells and higher numbers of Tregs compared with IRs, leading to a statistically significant reduction in the Th17/Treg ratio (71). Notably, this ratio correlates positively with intestinal tight junction protein ZO-1 expression and, within the gut mucosa, positively correlates with CD4^+^ T cell absolute counts and inversely correlates with HIV proviral DNA burden. Collectively, these findings indicate that Th17/Treg imbalance specifically localized to the intestinal mucosa represents a hallmark of incomplete immune reconstitution following ART. Furthermore, the presence of fibroblasts in INRs indicates that the intestinal mucosa has undergone significant fibrotic remodeling during the regeneration process (70). Therefore, persistent structural and functional impairment of the intestinal mucosal barrier likely represents a central pathological mechanism underlying immune non-response following ART.

It is noteworthy that several studies have reported an elevated proportion of Th17 cells in peripheral blood mononuclear cells among INRs, a finding that may reflect persistent immune activation or dysregulated T cell differentiation in the context of impaired overall CD4^+^ T cell recovery (97, 98). The proportion of CD4^+^ T cells with gut-homing characteristics in the peripheral blood of INRs was higher than that of IRs (22). The depletion of CD4^+^ T cells in INRs was widespread. Importantly, the proportion of depleted mucosal CD4^+^ T cells is positively correlated with circulating levels of intestinal epithelial damage markers, such as I-FABP and REG3α, indicating a potential association between CD4^+^ T cell loss and impaired intestinal barrier function. Compared with IRs, INRs exhibited significantly higher expression of integrin α4β7 on peripheral lymphocytes, with this upregulation observed across multiple lymphocyte subsets, including CD4^+^ T cells, CD8^+^ T cells, and B lymphocytes (99). Further analysis revealed that the frequencies of β7-expressing Th17 cells and Tregs were markedly elevated in INRs relative to both IRs and healthy controls (99). These findings suggest that α4β7-mediated gut homing may be aberrantly enhanced in distinct functional T cell subsets, potentially contributing to the impaired immune reconstitution observed in INRs. Compared with IRs, 3326 differentially expressed genes (DEGs) were identified in colon tissue from individuals with INRs, whereas no significantly differentially expressed genes were detected in ileal tissue (100). These DEGs in the colon of INRs were predominantly enriched in pathways associated with immune response regulation, metabolic processes, and cellular functional modulation. Notably, genes involved in B cell-mediated immunity and adaptive immune responses exhibited downregulation in the INR group. These findings indicate that incomplete immune reconstitution in PLWH following antiretroviral therapy may be closely linked to persistent molecular abnormalities in the sigmoid colon microenvironment. In another study, DEGs between IRs and INRs were predominantly enriched in pathways related to mitochondrial function and ribosomal biogenesis (101). Furthermore, INRs exhibited reduced proportions of Th1, Th17, and T follicular helper cells, along with upregulated markers of immune activation and T cell exhaustion. Flow cytometry analysis revealed decreased CD4^+^ T cell counts, elevated PD-1 and HLA-DR expression levels, and a reduction in resting memory B cell numbers, indicating persistent immune dysfunction. Single-cell RNA sequencing of colon tissue from individuals with INRs versus IRs revealed a significantly elevated proportion of B cells in INRs—particularly follicular B cells and memory B cells—whereas plasma cell abundance was markedly reduced (102). Moreover, in a cross-sectional study, INRs exhibited a higher rate of spontaneous activation than IRs in monocytes (as assessed by HLA-DR expression) and in both myeloid and plasmacytoid dendritic cells (as assessed by HLA-DR, CD83, and CD86 expression) (103). Therefore, given the multifactorial nature of immune dysfunction in INRs, in addition to ART, complementary auxiliary strategies are required to restore T cell and B cell function, thereby more effectively promoting immune reconstitution.

Interestingly, a study has shown a correlation between CD4^+^ T cells and intestinal microbiota. Recent and baseline CD4^+^ T cell counts were positively associated with the relative abundance of *Butyricimonas* and *Parabacteroides* and negatively associated with *Veillonella* and *Rothia* (5). The abundance of *Ruminococcaceae* and *Alistipes* was positively correlated with both nadir and current CD4^+^ T cell counts, and negatively correlated with CD8^+^CD57^+^ T cell counts; in contrast, *Roseburia* and *Blautia* showed negative correlations with nadir CD4^+^ T cell counts but positive correlations with CD8^+^CD57^+^ T cell counts (55). Therefore, intervention strategies targeting the modulation of intestinal dysbiosis hold promise as a novel approach to improving incomplete immune reconstitution in PLWH.

#### 4.4.2 MAIT cells in INRs

Mucosal-associated invariant T (MAIT) cells are a subset of innate-like T lymphocytes characterized by unique antigen recognition mechanisms and critical immune functions (104). Upon activation, MAIT cells secrete a range of cytokines (such as IFN-γ, TNF-α, and IL-17) and cytotoxic effector molecules (including perforin and granzymes). They thereby contribute to host defense against bacterial, fungal, and other microbial pathogens via both TCR-dependent and TCR-independent mechanisms (104, 105). Moreover, MAIT cells play a pivotal role in maintaining intestinal barrier integrity and regulating microbial homeostasis, and may influence the immune reconstitution process in PLWH (106, 107). In the early stages of HIV infection, intestinal MAIT cell numbers decline rapidly and profoundly, and this loss persists even after prolonged ART, with only partial recovery observed. Therefore, in-depth investigation of the phenotype, functional capacity, and microenvironmental regulation of intestinal MAIT cells in INRs may help elucidate the underlying mechanisms of impaired immune reconstitution and poor clinical outcomes (106). Comprehensive analysis of global transcriptional profiles in peripheral blood immune cells from ART-naïve PLWH, including IRs and INRs, has further revealed that MAIT cell dysfunction is closely associated with the host’s failure to control bacterial co-infections in INRs (108). Despite effective viral suppression following initiation of ART, MAIT cell numerical recovery remains incomplete, and functional immune restoration lags significantly, suggesting that the damage may be partially irreversible or require extended time for repair. In recent years, MAIT cells have garnered increasing attention as potential biomarkers of mucosal immune homeostasis and systemic inflammation, offering novel insights and therapeutic avenues for improving immune reconstitution in INRs.

#### 4.4.3 CD4/CD8 ratio in INRs

Prior to HIV-induced depletion of CD4^+^ T cells, CD8^+^ T cells in peripheral blood are typically elevated as a result of immune activation, leading to a decline in the CD4/CD8 ratio (109). Furthermore, a persistently low CD4/CD8 ratio has been significantly associated with increased AIDS-related mortality (110). One report showed that the nadir CD4^+^ T cell counts, current CD4^+^ T cell counts, and CD4/CD8 ratio were lower in the INRs group than in the IRs group, and the proportion of CD8^+^CD57^+^ T cells was also significantly reduced in the INRs group (55). Therefore, the CD4/CD8 ratio can be used as an important prognostic marker for the progression of PLWH and has potential clinical transformation value.

#### 4.4.4 Pro-inflammatory cytokines in INRs

Elevated pro-inflammatory cytokine levels represent a core mechanism underlying the persistence of chronic inflammation in HIV/AIDS and are especially pronounced in INRs. Inflammatory markers (IL-2, IL-4, IL-6, IL-10, IFN-γ, and TNF-α) were higher in the INRs and IRs groups than in the healthy control group, but there were no significant differences between the INRs and IRs groups (55). In another study, compared with IRs, INRs exhibited significantly elevated serum levels of TNF-α, interferon-γ-inducible protein (IP)-10, and interleukin-1α (IL-1α) (47). Furthermore, CD4^+^ T cell counts were negatively correlated with TNF-α and IL-1α levels and positively correlated with the relative abundance of *Ruminococcaceae* (47). Therefore, lower CD4^+^ T cell counts in INRs are closely associated with reduced intestinal abundance of *Ruminococcaceae* and elevated serum levels of pro-inflammatory cytokines. Given this association, therapeutic interventions targeting the gut microbiota to restore CD4^+^ T cell counts represent a promising strategy for promoting immune reconstitution in HIV-infected individuals with incomplete immunological recovery.

## 5 Therapeutic strategies for INRs targeting the gut microbiota

The synergistic interaction between intestinal dysbiosis and intestinal mucosal immune dysfunction drives persistent abnormal immune activation, thereby significantly impairing CD4^+^ T cell recovery in individuals with INRs. In addition, individuals with INRs consistently exhibit a significant reduction in gut microbiota alpha diversity, indicative of microbial ecological imbalance. Consequently, enhancing intestinal epithelial barrier function and implementing targeted modulation of microbiota composition represent promising therapeutic strategies to ameliorate associated immune dysfunction (**Table 2**).

**Table 2:** Therapeutic strategies for INRs targeting the gut microbiota.

Drugs	Number of patients	Specific mechanisms	Ref.
Predigested enteral nutrition supplements	59	Increase the number of CD4^+^ T cells, but also reduce the levels of DAO, D-lactate and LPS	(111)
*Lactobacillus plantarum* and *Pediococcus acidilactici*	71	Increase the CD4/CD8 ratio and reduce sCD14 levels	(112)
Wenshen Jianpi recipe	56	Increase the CD4^+^ T cells and reduce CD56negCD16^+^ NK cells	(113)
(5R)-5-hydroxytriptolide	149	Increase CD4^+^ T cell counts and reduce IP-10 levels	(114)
*Saccharomyces boulardii*	44	Reduce *Clostridiaceae* abundance and lowers levels of IL-6 and LBP	(115, 116)
Probiotic capsule	20	Increase ileal microbial alpha diversity, enriched *Bifidobacterium* abundance, and elevate the CD4/CD8 ratio	(117)
Metformin	–	Attenuation of T cell exhaustion, suppression of systemic chronic inflammation, and partial restoration of gut microbiota composition disrupted	(121)

DAO, diamine oxidase; LPS, lipopolysaccharide; IP-10, interferon-γ-induced protein 10; LBP, lipopolysaccharide-binding protein.

A study has shown that oral predigested enteral nutrition supplements significantly increase both CD4^+^ and CD8^+^ T cell counts while concurrently reducing serum levels of diamine oxidase (DAO), D-lactate, and LPS, suggesting that such enteral nutrition intervention helps restore intestinal barrier integrity and represents a promising strategy for promoting immune reconstitution in INRs (111). A double-blind, placebo-controlled clinical trial demonstrated that a probiotic mixture containing *Lactobacillus plantarum* and *Pediococcus acidilactici* significantly increased the CD4/CD8 ratio and reduced sCD14 levels in PLWH; however, the clinical relevance of these immunomodulatory effects remains uncertain and warrants validation in larger, longer-term trials (112). Moreover, the Wenshen Jianpi recipe has been demonstrated to significantly increase the frequency of CD4^+^ T cells and reduce the proportion of CD56negCD16^+^ NK cells in INRs (113). Another clinical trial demonstrated that (5R)-5-hydroxytriptolide significantly promoted the recovery of peripheral blood CD4^+^ T cell counts and reduced IP-10 levels in INRs (114). Additionally, the probiotic Saccharomyces boulardii significantly reduced *Clostridiaceae* relative abundance in INRs and concurrently lowered circulating levels of IL-6 and lipopolysaccharide-binding protein (LBP) (115, 116). Probiotic capsule administration significantly increased ileal microbial alpha diversity, enriched *Bifidobacterium* abundance, and elevated the peripheral blood CD4/CD8 ratio in INRs; however, it did not significantly affect total body CD4^+^ T cell counts (117). However, relevant clinical research is still limited and lacks sufficient evidence to confirm this. There is an urgent need to conduct more high-quality clinical trials to verify these findings. However, another clinical trial did show that PMT25341—a mixture of synbiotics, omega-3/6 fatty acids, and amino acids—did not improve average CD4^+^ T cell counts or the CD4/CD8 ratio in INRs (118). At the same time, another probiotic, *Lactobacillus casei Shirota*, did not affect immune activation markers, CD4^+^ and CD8^+^ T cell subsets, plasma sCD14 levels, or NK cell frequency; nor did it improve gut microbiota composition (119). The failure of relevant probiotic intervention to achieve the expected improvement may be due to the fact that the supplemented strain is not the key beneficial bacteria specifically lacking in the intestines of INRs. Therefore, precise probiotic intervention strategies should be formulated based on the personalized microflora profile characteristics revealed by metagenome or 16S rRNA sequencing to target correction of intestinal flora disorders in INRs and thereby support their immune reconstruction process. Metformin, a widely prescribed oral antihyperglycemic agent, has emerged in recent years as a candidate immunomodulator (120). Clinical evidence indicates that it may modestly reduce the size of the HIV reservoir and enhance CD4^+^ T cell count recovery. Mechanistic studies suggest that these effects are potentially mediated through attenuation of T cell exhaustion, suppression of systemic chronic inflammation, and partial restoration of gut microbiota composition disrupted by HIV infection (121, 122). Accordingly, metformin is considered a promising repurposed therapeutic candidate for improving immune reconstitution in individuals with INRs.

## 6 Conclusions and future directions

### 6.1 Dysregulation of the intestinal microenvironment in INRs contributes to incomplete immune reconstitution

HIV/AIDS is a major challenge facing global public health. Although ART can effectively inhibit HIV replication, significantly prolong patient survival, and improve quality of life, some patients fail to effectively recover CD4^+^ T cell counts after long-term treatment, resulting in poor clinical prognosis and an increased social and medical burden. This review points out that patients with INRs generally exhibit more significant intestinal dysbiosis and immune dysfunction, manifested by sustained CD4^+^ T cell depletion, reduced Th17 cell counts, impaired intestinal barrier function (e.g., downregulation of ZO-1 and claudin-1), increased microbial translocation (e.g., elevated LPS levels), persistent chronic inflammation, and intestinal microbiota imbalance. These pathological characteristics are closely associated with disease progression and an increased risk of opportunistic infections, and they also provide a theoretical basis for adjuvant treatment strategies targeting the intestinal immune microenvironment. Intestinal dysbiosis is one of the main mechanisms underlying impaired immune reconstitution in INRs. In-depth exploration of how the intestinal microbiota influences immune reconstitution at the molecular level will enhance understanding of the pathogenesis of INRs and provide a theoretical basis for optimizing clinical treatment strategies and developing novel therapeutics. However, existing research is limited by methodological bottlenecks, particularly the lack of reliable animal models that can simultaneously recapitulate the hallmark features of INRs—including intestinal dysbiosis, mucosal immune deficiency, and systemic inflammation—which severely restricts the depth and credibility of mechanistic validation. These findings suggest that targeted interventions against local mucosal immune dysregulation and colonic tissue injury may ameliorate defective immune reconstitution and represent a promising therapeutic strategy to reduce the risk of long-term complications and mortality in patients with INRs.

### 6.2 Future research priorities

Multiple studies have demonstrated that antiretroviral drugs can directly disrupt intestinal microbial homeostasis (123–125). For instance, zidovudine and efavirenz significantly inhibit the growth of *Bacteroides fragilis* and *Prevotella in vitro*; efavirenz has also been shown to suppress the proliferation of *Enterococcus faecalis* (123). Consequently, ART not only fails to restore the intestinal dysbiosis induced by HIV infection but may also further exacerbate both structural imbalances and functional impairments of the gut microbiota—partly attributable to its intrinsic broad-spectrum antibacterial activity. Notably, in contrast, the integrase strand transfer inhibitor dolutegravir has been associated with increased alpha diversity and species richness of the gut microbiota, suggesting a potential restorative effect on microbial ecology (124). In summary, the development of a novel ART strategy—integrating potent antiviral efficacy with preservation of intestinal microbial homeostasis—is expected to represent a pivotal advancement in PLWH treatment.

Probiotic and prebiotic interventions have shown clear application value in enhancing immune function and regulating the composition and function of the intestinal microbiota. Studies have shown that probiotic supplementation can improve intestinal barrier function, modulate microbial composition, reduce microbial translocation, and enhance intestinal mucosal immunity in PLWH (126–128). However, current research still faces several limitations: First, most existing evidence comes from cross-sectional or observational studies, and their inherent design limitations impede causal inference; second, randomized controlled trials targeting probiotic and synbiotic interventions generally suffer from small sample sizes and insufficient follow-up durations, resulting in a lack of high-quality, evidence-based support for the persistence, stability, and long-term safety of intervention effects; finally, baseline differences in the gut microbiome among individuals, heterogeneity of ART regimens, and lifestyle-related confounding factors—such as diet and stress—may all interfere with the consistent manifestation of intervention effects, thereby weakening the external validity and clinical generalizability of study findings. In the future, there is an urgent need to conduct rigorous, multicenter, large-sample scale prospective cohort studies and randomized controlled trials; to integrate multi-omics data— including metagenomics and metabolomics—and to incorporate precision medicine principles to identify biomarkers; thereby establishing an individualized nutritional intervention strategy based on microbiome profiling to effectively promote immune reconstitution in INRs.

Importantly, the current definition and diagnostic criteria for INRs differ significantly across studies, hindering data integration and knowledge accumulation and resulting in poor comparability among study findings. There is an urgent need for international authoritative organizations to establish unified definitions and standards to promote standardized research and data integration for this condition, thereby providing robust evidence to support immune reconstitution in INRs, improve their clinical outcomes, and reduce mortality.

## Glossary

AIDS: Acquired immunodeficiency syndrome
ART: antiretroviral therapy
CCR5: C-C motif chemokine receptor 5
DAO: diamine oxidase
DEGs: differentially expressed genes
GALT: gut-associated lymphoid tissue
GT25: Glycosyltransferase 25
HIV: human immunodeficiency virus
I-FABP: intestinal fatty acid-binding protein
IP-10: interferon-γ-inducible protein-10
INRs: immunological non-responders
LBP: lipopolysaccharide-binding protein
LPS: lipopolysaccharide
MAIT: Mucosal-associated invariant T
PLWH: people living with HIV
REG3α: regenerating islet-derived protein 3 alpha
SCFAs: short-chain fatty acids
sCD14: soluble CD14
